# Sulfadoxine-Pyrimethamine Exhibits Dose-Response Protection Against Adverse Birth Outcomes Related to Malaria and Sexually Transmitted and Reproductive Tract Infections

**DOI:** 10.1093/cid/cix026

**Published:** 2017-03-02

**Authors:** R. Matthew Chico, Enesia Banda Chaponda, Cono Ariti, Daniel Chandramohan

**Affiliations:** 1 Department of Disease Control, London School of Hygiene & Tropical Medicine, United Kingdom;; 2 Department of Biological Sciences, University of Zambia, Lusaka; and; 3 Department of Medical Statistics, London School of Hygiene & Tropical Medicine, United Kingdom

**Keywords:** malaria, curable sexually transmitted infections, antenatal care, intermittent preventive treatment, sub-Saharan Africa.

## Abstract

**Background.:**

We conducted a prospective cohort study in Zambia among pregnant women who received intermittent preventive treatment using sulfadoxine-pyrimethamine (IPTp-SP).

**Methods.:**

We calculated the odds ratios (ORs) of adverse birth outcomes by IPTp-SP exposure, 0–1 dose (n = 126) vs ≥2 doses (n = 590) and ≥2 doses (n = 310) vs ≥3 doses (n = 280) in 7 categories of malaria infection and sexually transmitted and reproductive tract infections (STIs/RTIs).

**Results.:**

We found no significant differences in baseline prevalence of infection across IPTp-SP exposure groups. However, among women given 2 doses compared to 0–1 dose, the odds of any adverse birth outcome were reduced 45% (OR, 0.55; 95% confidence interval [CI], 0.36, 0.86) and 13% further with ≥3 doses (OR, 0.43; 95% CI, 0.27, 0.68). Two or more doses compared to 0–1 dose reduced preterm delivery by 58% (OR, 0.42; 95% CI, 0.27, 0.67) and 21% further with ≥3 doses (OR, 0.21; 95% CI, 0.13, 0.35). Women with malaria at enrollment who received ≥2 doses vs 0-1 had 76% lower odds of any adverse birth outcome (OR, 0.24; 95% 0.09, 0.66), and *Neisseria gonorrhoeae* and/or *Chlamydia trachomatis* had 92% lower odds of any adverse birth outcome (OR, 0.08; 95% CI, 0.01, 0.64). Women with neither a malaria infection nor STIs/RTIs who received ≥2 doses had 73% fewer adverse birth outcomes (OR, 0.27; 95% CI, 0.11, 0.68).

**Conclusions.:**

IPTp-SP appears to protect against malaria, STIs/RTIs, and other unspecified causes of adverse birth outcome.

## Summary

Compared to 0–1 dose of intermittent preventive treatment of malaria using sulfadoxine-pyrimethamine, ≥2 doses protects pregnant women against adverse birth outcomes attributable to malaria infection and to curable sexually transmitted and reproductive tract infections.

To reduce the adverse consequences of malaria infection during pregnancy, the World Health Organization (WHO) recommends administering sulfadoxine-pyrimethamine (SP) as intermittent preventive treatment (IPTp) to women during every scheduled antenatal care (ANC) visit at least 1 month apart during the second trimester and at delivery in areas of moderate to high malaria transmission [1]. Although the WHO recommendation for IPTp-SP does not extend to low-transmission settings, a recent metaregression analysis suggests there is not a threshold of malaria transmission intensity below which ≥2 doses of IPTp-SP are no longer protective against the incidence of low birth weight (LBW) in sub-Saharan Africa [2]. One possible reason could be that IPTp-SP is protective against malaria as well as other nonmalarial causes of LBW. Five curable sexually transmitted and reproductive tract infections (STIs/RTIs)—syphilis, *Neisseria gonorrheae*, *Chlamydia trachomatis*, *Trichomonas vaginalis*, and bacterial vaginosis—are associated with several adverse birth outcomes that include stillbirth [3–5], LBW [4–9], preterm birth [5, 8, 10–13], and intrauterine growth retardation (IUGR) [4, 5]. Thus, we analyzed data from a prospective cohort of pregnant women, relating the incidence of malaria infection and curable STIs/RTIs, maternal exposure to IPTp-SP during the antenatal period (0–1 doses vs ≥2 doses and, separately, 2 doses vs ≥3 doses), and the resulting incidence of the following 4 adverse birth outcomes: stillbirth, LBW, preterm delivery, and IUGR.

## METHODS

We recruited 1086 pregnant women between November 2013 and April 2014 on their first ANC visit to health centers operated by the Ministry of Health in the Nchelenge District of Zambia as described elsewhere [14, 15]. Nchelenge is in the Luapula province 290 km (180 miles) from Mansa, the provincial capital, and along the shores of Lake Mweru in northeastern Zambia. Gestational age was measured at enrollment to determine eligibility for participation and was based on last menstrual period, symphysis-fundal height, and sonography. Women who were not already known to have AIDS were tested for human immunodeficiency virus (HIV) per ministry practices at the time. HIV-infected women were given triple antiretroviral therapy if their CD4 count was <500 cells/mm^3^ of blood, a variation on “option B” treatment originally recommended by the WHO in 2010 [16]. Study staff tested women for syphilis using rapid plasma reagin (RPR) methods. If positive, women received a written notice to return to the health facility with their partners for treatment. Biological samples for malaria and other curable STIs/RTIs were collected at enrollment and transported to a reference laboratory for retrospective batch-testing using polymerase chain reaction techniques. Placental samples were collected at delivery for malaria histology. Women were given IPTp-SP during scheduled ANC visits and followed through delivery to record birth outcomes. Women were encouraged to deliver at a health facility to simplify data collection. If willing to do so, the women were provided free transport to and from the only hospital in the district, Saint Paul’s Mission Hospital, or Kashikishi Health Centre; Nchelenge Health Center did not offer maternity services at the time. We analyzed data using Stata, version 13 (StataCorp, College Station, Texas) software.

### Descriptive Analysis

We summarized categorical variables as whole numbers and percentages and continuous variables by means and standard deviations if the data appeared to be normally distributed. We then assessed the differences in the characteristics of participants at the time of enrollment between women who had received 0–1 dose vs ≥2 doses and, separately, 2 doses vs ≥ 3 doses, using Wilcoxon test for continuous variables and Fisher exact test for categorical variables. We then calculated the crude odds ratios (ORs) of adverse birth outcomes among pregnant women who received 0–1 dose compared to ≥2 doses and 2 doses compared to ≥3 doses with logistic regression models.

### Assessment of Confounding and Effect Modification

At the onset, we considered HIV status and gravidae to be confounders on the protective effect of IPTp-SP against adverse birth outcomes based on evidence in the public domain [17, 18]. We used common statistical methods to identify other potential confounders [19], entering variables into a logistic regression model that included categories of IPTp-SP exposure. We considered variables to be confounders a priori if they produced a change in ORs between crude and adjusted by ≥10% (Supplementary Tables 2–6). We tested effect modification by adding an interaction term between these potential confounders and the number of IPTp-SP doses and then applied a likelihood ratio test to see if there was evidence of interaction. We observed some evidence of effect modification with malaria and ST/RT coinfection, which we dealt with separately as described below. We also considered malaria and ST/RT coinfection as a confounder. We then entered all confounding variables into a multivariable logistic regression model to estimate overall adjusted ORs.

### Calculation of Coinfection Odds Ratios

We examined the distribution of infection and constructed the following 7 mutually exclusive maternal infection categories: malaria only, malaria and *N. gonorrhoeae* and/or *C. trachomatis*, malaria and *T. vaginalis* and/or bacterial vaginosis, syphilis and any other infection(s), *N. gonorrhoeae, C. trachomatis* only, *T. vaginalis* and/or bacterial vaginosis only, and no identified infection. We then calculated stratum-specific ORs of IPTp-SP exposure for each infection category using a multivariable logistic regression model that included all potential confounders as well as interaction terms.

## RESULTS

We found no significant differences in the prevalence of malaria infection and curable STIs/RTIs at baseline across IPTp-SP exposure groups stratified by 0–1 dose (n = 126) vs ≥2 doses (n = 590; Table 1) and, separately, 2 doses (n = 310) vs ≥3 doses (n = 280) (Supplementary Table 1). However, the odds of any adverse birth outcome among women who received ≥2 doses compared to 0–1 dose (Table 2) were reduced 45% (OR, 0.55; 95% confidence interval [CI], 0.36, 0.86) and 12% further (57% total reduction) with ≥3 doses (OR, 0.43; 95% CI, 0.27, 0.68). Two or more doses compared to 0–1 dose reduced the odds of preterm delivery by 58% (OR, 0.42; 95% CI, 0.27, 0.67) and 21% further (79% total reduction) with ≥3 doses (OR, 0.21; 95% CI, 0.13, 0.35; Table 2).

**Table 1.** Participant Characteristics by Exposure to 0–1 Dose vs ≥ 2 Doses of Intermittent Preventive Treatment Against Malaria Using Sulfadoxine-Pyrimethamine

**Characteristic at Enrollment**	**Doses of Sulfadoxine-Pyrimethamine Received, no. (%**)^**a**^				***P* Value** ^**b**^
	**0–1 Dose**		**≥2 Doses**		
	**n = 126**		**n = 590**		
Age of participants					.498
Mean (standard deviation)	25.8	(6.5)	25.4	(6.4)	
Median (interquartile range)	24.0	(20.0, 31.0)	24.0	(20.0, 30.0)	
Marital status					.175
Single	19	(15.1)	123	(20.8)	
Married, divorced/separated, or widowed	107	(84.9)	467	(79.2)	
Age at sexual debut, y					.733
<15	13	(10.3)	49	(8.3)	
≥15	96	(76.2)	455	(77.1)	
Unknown	17	(13.5)	86	(14.6)	
Number of lifetime sexual partners					.362
1	52	(41.3)	272	(46.6)	
2	45	(35.7)	161	(27.6)	
3	18	(14.3)	94	(16.1)	
4 or more	11	(8.7)	57	(9.8)	
Gravidae					.301
Primigravidae	27	(21.4)	165	(28.0)	
Secundigravidae	19	(15.1)	77	(13.1)	
Multigravidae	80	(63.5)	348	(59.0)	
Wealth quintiles					.048
Lowest	21	(16.7)	115	(19.5)	
Second	32	(25.4)	111	(18.8)	
Middle	31	(24.6)	113	(19.2)	
Fourth	14	(11.1)	122	(20.7)	
Highest	28	(22.2)	129	(21.9)	
Bed net ownership					.493
No	68	(54.0)	297	(50.3)	
Yes	58	(46.0)	293	(49.7)	
Used insecticide-treated net on previous night					.840
No	77	(61.1)	366	(62.4)	
Yes	49	(38.9)	221	(37.6)	
Missing	0		3		
Indoor residual spraying in the previous 12 months					.186
No	103	(83.1)	439	(77.6)	
Yes	21	(16.9)	127	(22.4)	
Missing	2		24		
Experienced miscarriage before					.869
No	86	(86.9)	371	(87.3)	
Yes	13	(13.1)	54	(12.7)	
None reported by primigravidae	27		165		
Delivered a premature baby before					1.000
No	94	(94.9)	401	(94.4)	
Yes	5	(5.1)	24	(5.6)	
Not applicable to primigravidae	27		165		
Delivered a stillborn before					.307
No	94	(94.9)	387	(91.1)	
Yes	5	(5.1)	38	(8.9)	
Not applicable to primigravidae	27		165		
Human immunodeficiency virus status					.186
Negative	105	(83.3)	519	(88.0)	
Positive	21	(16.7)	71	(12.0)	
Malaria and curable STIs/RTIs					
Malaria (polymerase chain reaction diagnosis)	62	(49.2)	346	(59.3)	.047
Syphilis (high titer)	1	(0.8)	17	(2.9)	.223
*Neisseria gonorrhoeae*	1	(0.8)	21	(3.6)	.152
*Chlamydia trachomatis*	8	(6.3)	26	(4.4)	.357
*Trichomonas vaginalis*	30	(23.8)	140	(23.7)	1.000
Bacterial vaginosis	59	(46.8)	277	(46.9)	1.000
**Characteristics at Delivery**					
Place of delivery					.233
Hospital	119	(94.4)	551	(93.4)	
Clinic	1	(0.8)	19	(3.2)	
Home	6	(4.8)	20	(3.4)	
Delivery performed by					.240
Doctor	3	(2.4)	38	(6.4)	
Midwife	115	(91.3)	524	(88.8)	
Family member	5	(4.0)	17	(2.9)	
Other	3	(2.4)	11	(1.9)	
Type of labor					.296
Spontaneous	126	(100.0)	558	(97.4)	
Induced	0	(0.0)	9	(1.6)	
Augmented	0	(0.0)	6	(1.0)	
Type of delivery					.092
Vaginal	123	(97.6)	551	(93.4)	
Cesarean section	3	(2.4)	39	(6.6)	
Hypertension					.296
No	110	(96.5)	506	(98.1)	
Yes	4	(3.5)	10	(1.9)	
Maternal hemoglobin					.786
Normal	103	(85.1)	470	(83.5)	
Anemic	18	(14.9)	93	(16.5)	
Sex of baby					.008
Female	78	(61.9)	287	(48.6)	
Male	48	(38.1)	303	(51.4)	
Received curative treatment for malaria infection					.102
No	115	(92.0)	508	(86.4)	
Yes	10	(8.0)	80	(13.6)	
Received curative treatment for any STI/RTI					1.000
Untreated	116	(92.1)	540	(91.5)	
Treated	10	(7.9)	50	(8.5)	

^a^Age is shown as the median value with the interquartile range in parentheses.

^b^ *P* values are from Wilcoxon rank sum test (continuous variables) or Fisher exact test (categorical variables).

Abbreviations: STI, sexually transmitted infections; RTI, reproductive tract infections.

**Table 2.** Adverse Birth Outcomes by Exposure to 0–1 Dose vs 2 Doses vs ≥ 3 Doses of Intermittent Preventive Treatment Against Malaria Using Sulfadoxine-Pyrimethamine

**Birth Outcome**	**No. of Women**	**Outcomes**	**Unadjusted OR**	**95% CI**	**Adjusted OR** ^a^	**95% CI**	***P* Value** ^b^
Any adverse outcome							
0–1 dose	126	58	1.00		1.00		**.002**
2 doses	310	108	0.63	0.41, 0.96	0.55	**0.36, 0.86**	
≥3 doses	280	84	0.50	0.33, 0.78	0.43	**0.27, 0.68**	
Stillbirth							
0–1 dose	126	4	1.00		1.00		.143
2 doses	310	2	0.20	0.04, 1.10	0.21	0.04, 1.19	
≥3 doses	280	6	0.67	0.19, 2.41	0.68	0.18, 2.57	
Low birth weight							
0–1 dose	126	32	1.00		1.00		.261
2 doses	310	67	0.80	0.49, 1.30	0.71	0.42, 1.19	
≥3 doses	280	57	0.74	0.45, 1.22	0.64	0.37, 1.09	
Preterm delivery							
0–1 dose	126	50	1.00		1.00		**<0.001**
2 doses	310	71	0.45	0.29, 0.71	0.42	**0.27, 0.67**	
≥3 doses	280	37	0.23	0.14, 0.38	0.21	**0.13, 0.35**	
Intrauterine growth retardation							
0–1 dose	126	7	1.00		1.00		.318
2 doses	310	34	1.64	0.70, 3.87	1.55	0.64, 3.77	
≥3 doses	280	43	2.12	0.91, 4.93	1.88	0.78, 4.54	

CIs that do not overlap the null value of OR = 1 are shown in bold.

Abbreviations: CI, confidence interval; OR, odds ratio.

^a^Adjusted for sexually transmitted and reproductive tract coinfection, gravidae, and human immunodeficiency virus coinfection.

^b^ *P* value for likelihood ratio test.

This dose-response relationship was also observed against malaria infection and STIs/RTIs in IPTp-SP dosing categories of 0–1 vs ≥2 doses and 2 vs ≥ 3 doses. In 24 of 30 infection categories, ≥2 doses conferred greater protection against adverse birth outcomes than 0–1 dose, 8 of which showed significant effects (Table 3); 3 categories related to any birth outcome, 1 was specific to LBW, and 4 were associated with preterm birth.

**Table 3.** Categories of Maternal Infection and Exposure to Dose 0–1 vs ≥ 2 Doses of Intermittent Preventive Treatment Against Malaria Using Sulfadoxine-Pyrimethamine Among Women with Adverse Birth Outcomes

**Adverse Birth Outcome**	**0–1 dose IPTp-SP**		**≥2 doses IPTp-SP**					
**Categories of Maternal Infection**	**No. of Women**	**No. of Outcomes**	**No. of Women**	**No. of Outcomes**	**Crude OR**	**95% CI**	**Adjusted OR** ^a^	**95% CI**
Any adverse outcome								
Malaria only	20	13	129	41	0.25	0.09, 0.88	**0.24**	**0.09, 0.66**
Malaria and NG and/or CT	3	1	27	11	1.38	0.11, 17.09	1.17	0.09, 15.89
Malaria and TV and/or BV	38	15	182	67	0.89	0.44, 1.83	0.96	0.45, 2.02
Syphilis and any other infection(s)^b^	1	1	17	7	0.80	0.00, 31.20	0.80	0.00, 31.20
NG and/or CT only	6	4	14	2	0.08	0.01, 0.80	**0.08**	**0.01, 0.64**
TV and/or BV only	32	12	124	42	0.85	0.38, 1.91	0.72	0.32, 1.65
No identified infection	26	12	97	22	0.34	0.14, 0.85	**0.27**	**0.11, 0.68**
Stillbirth								
Malaria only	20	1	129	1	0.15	0.01, 2.54	0.15	0.01, 2.54
Malaria and NG and/or CT	3	0	27	0	NA	NA	NA	NA
Malaria and TV and/or BV	38	0	182	3	0.81	0.09, Inf	0.81	0.09, Inf
Syphilis and any other infection(s)	1	1	17	0	0.06	0.00, 2.29	0.06	0.00, 2.29
NG and/or CT only	6	0	14	0	NA	NA	NA	NA
TV and/or BV only	32	1	124	3	0.76	0.07, 7.86	0.76	0.07, 7.86
No identified infection	26	1	97	1	0.26	0.02, 4.31	0.22	0.01, 3.79
Low birth weight								
Malaria only	20	6	129	25	0.56	0.20, 1.60	0.59	0.19, 1.82
Malaria and NG and/or CT	3	1	27	7	0.7	0.05, 8.97	0.49	0.03, 7.35
Malaria and TV and/or BV	38	10	182	46	0.95	0.43, 2.10	1.08	0.46, 2.54
Syphilis and any other infection(s)	1	0	17	5	NA	NA	NA	NA
NG and/or CT only	6	2	14	1	0.15	0.01, 2.18	0.12	0.01, 1.90
TV and/or BV only	32	5	124	27	1.5	0.53, 4.27	1.22	0.41, 3.59
No identified infection	26	8	97	13	0.35	0.13, 0.96	**0.24**	**0.08, 0.68**
Preterm delivery								
Malaria only	20	10	129	21	0.19	0.07, 0.53	**0.19**	**0.07, 0.53**
Malaria and NG and/or CT	3	0	27	6	NA	NA	NA	NA
Malaria and TV and/or BV	38	14	182	37	0.44	0.21, 0.93	**0.45**	**0.21, 0.97**
Syphilis and any other infection(s)	1	1	17	5	0.50	0.00, 19.50	0.50	0.00, 19.50
NG and/or CT only	6	4	14	2	0.08	0.01, 0.80	**0.07**	**0.01, 0.73**
TV and/or BV only	32	11	124	25	0.48	0.21, 1.13	0.43	0.18, 1.03
No identified infection	26	10	97	12	0.23	0.08, 0.61	**0.20**	**0.07, 0.54**
Intrauterine growth retardation								
Malaria only	20	2	129	18	0.8	0.16, 4.08	0.75	0.14, 4.05
Malaria and NG and/or CT	3	1	27	5	0.62	0.05, 8.43	0.54	0.03, 8.43
Malaria and TV and/or BV	38	1	182	27	5.35	0.69, 41.40	6.11	0.76, 49.13
Syphilis and any other infection(s)	1	0	17	2	NA	NA	NA	NA
NG and/or CT only	6	0	14	0	NA	NA	NA	NA
TV and/or BV only	32	1	124	16	3.81	0.48, 30.44	3.13	0.38, 25.68
No identified infection	26	2	97	9	0.83	0.16, 4.25	0.66	0.12, 3.57

CIs that do not overlap the null value of OR = 1 are shown in bold. Syphilis and coinfection describes pregnant women who tested positive for syphilis using rapid plasma reagin assays and were also infected with malaria and/or another curable sexually transmitted or reproductive tract infection. NA (not applicable) is used where there are no observations in the reference group (0–1 dose). Inf (infinity) is used where the subsample of outcomes observed is too small to produce an outer limit with certainty.

Abbreviations: BV, bacterial vaginosis; CI, confidence interval; CT, *Chlamydia trachomatis*; IPTp-SP, intermittent preventive treatment of malaria in pregnancy using sulfadoxine-pyrimethamine; NG, *Neisseria gonorrhoeae*; OR, odds ratio; TV, *Trichomonas vaginalis*.

^a^Adjusted for sexually transmitted and reproductive tract coinfection, gravidae, and human immunodeficiency virus coinfection.

^b^Syphilis and any other infection(s) refers to any other curable sexually transmitted or reproductive tract infection and/or malaria.

Women who had malaria only at enrollment and received ≥2 doses of versus 0-1 had 76% lower odds of any adverse birth outcome (OR, 0.24; 95% 0.09, 0.66), whereas women who had *N. gonorrhoeae* and/or *C. trachomatis* at enrollment and were provided ≥2 doses of versus 0-1, had 92% lower odds of any adverse birth outcome (OR, 0.08; 95% CI, 0.01, 0.64). Women with neither a malaria infection nor STIs/RTIs and who received ≥2 doses, rather than 0–1 dose, had 73% fewer adverse birth outcomes (OR, 0.27; 95% CI, 0.11, 0.68). Similarly, women with neither a malaria infection nor STIs/RTIs and who received ≥2 doses had the odds of LBW reduced by 76% (OR, 0.24; 95% CI, 0.08, 0.68).

Women who had malaria only at enrollment and received ≥2 doses of versus 0-1, had 81% lower odds of preterm birth (OR, 0.19; 95% CI, 0.07, 0.53), whereas participants who had malaria plus *T. vaginalis* and/or bacterial vaginosis at enrollment and received ≥2 doses of versus 0-1, had 55% lower odds of preterm birth (OR, 0.45; 95% CI, 0.21, 0.97). In the same way, women who had *N. gonorrhoeae* or *C. trachomatis* at enrollment and received ≥2 doses of versus 0-1, had 93% lower odds of preterm birth (OR, 0.07; 95% CI, 0.01, 0.73), and those who had neither malaria nor any curable STI/RTI at enrollment and received ≥2 doses of versus 0-1, had 80% lower odds of preterm birth (OR, 0.20; 95% CI, 0.07, 0.54).

This dose-response effect extended, although less pronounced, in comparisons of outcomes following ≥3 doses vs 2 doses (Table 4). There appeared to be a trend of protection in 17 of 31 infection categories conferred by ≥3 doses vs 2 doses, but this protective effect was only significant in 2 infection categories (Table 4). Women who had malaria plus *T. vaginalis* and/or bacterial vaginosis at enrollment and received ≥3 doses vs 2 doses had 67% lower odds of preterm birth (OR, 0.33; 95% CI, 0.15, 0.73), whereas participants who had *T. vaginalis* and/or bacterial vaginosis at enrollment and received ≥3 doses vs 2 doses had 66% lower odds of preterm birth (OR, 0.34; 95% CI, 0.13, 0.94).

**Table 4.** Categories of Maternal Infection and Exposure to 2 Doses vs ≥ 3 Doses of Intermittent Preventive Treatment Against Malaria Using Sulfadoxine-Pyrimethamine Among Women With Adverse Birth Outcomes

**Adverse Birth Outcomes**	**2 Doses IPTp-SP**		**≥3 Doses IPTp-SP**					
**Categories of Maternal Infection**	**No. of Women**	**No. of Outcomes**	**No. of Women**	**No. of Outcomes**	**Crude OR**	**95% CI**	**Adjusted OR** ^a^	**95% CI**
Any adverse outcome								
Malaria only	66	23	63	18	0.75	0.35, 1.58	0.72	0.33, 1.56
Malaria and NG and/or CT	18	7	9	4	1.26	0.25, 6.36	0.93	0.17, 5.16
Malaria and TV and/or BV	86	36	96	31	0.66	0.36, 1.21	0.66	0.35, 1.24
Syphilis and any other infection(s)^b^	7	3	10	4	0.89	0.13, 6.31	0.5	0.07, 3.72
NG and/or CT only	12	1	2	1	11	0.35, 345.05	16.39	0.50, 541.53
TV and/or BV only	71	27	53	15	0.64	0.30, 1.38	0.64	0.29, 1.41
No identified infection	50	11	47	11	1.08	0.42, 2.80	1.28	0.48, 3.43
Stillbirth								
Malaria only	66	1	63	0	1.03	0.00, 40.32	1.03	0.00, 40.32
Malaria and NG and/or CT	18	0	9	0	N/A	-	N/A	-
Malaria and TV and/or BV	86	1	96	2	1.81	0.09, 107.93	1.81	0.09, 107.93
Syphilis and any other infection(s)	7	0	10	0	N/A	-	N/A	-
NG and/or CT only	12	0	2	0	N/A	-	N/A	-
TV and/or BV only	71	0	53	3	5.32	0.56, Inf	5.32	0.56, Inf
No identified infection	50	0	47	1	1.81	0.16, 20.30	2.18	0.19, 25.65
Low birth weight								
Malaria only	66	14	63	11	0.79	0.33, 1.89	0.74	0.30, 1.86
Malaria and NG and/or CT	18	5	9	2	0.74	0.11, 4.87	0.49	0.07, 3.62
Malaria and TV and/or BV	86	23	96	23	0.86	0.44, 1.68	0.91	0.45, 1.86
Syphilis and any other infection(s)	7	2	10	3	1.07	0.13, 8.98	0.53	0.06, 4.87
NG and/or CT only	12	1	2	0	6.00	0.00, 234	6.00	0.00, 234
TV and/or BV only	71	16	53	11	0.90	0.38, 2.14	0.90	0.38, 2.14
No identified infection	50	6	47	7	1.28	0.40, 4.14	1.59	0.47, 5.40
Preterm delivery								
Malaria only	66	13	63	8	0.59	0.23, 1.55	0.59	0.22, 1.54
Malaria and NG and/or CT	18	4	9	2	1.00	0.15, 6.85	0.83	0.12, 5.82
Malaria and TV and/or BV	86	25	96	12	0.34	0.16, 0.74	**0.33**	**0.15, 0.73**
Syphilis and any other infection(s)	7	3	10	2	0.33	0.04, 2.87	0.23	0.03, 2.06
NG and/or CT only	12	1	2	1	11	0.35, 345.06	14.4	0.45, 463.93
TV and/or BV only	71	19	53	6	0.35	0.13, 0.95	**0.34**	**0.13, 0.94**
No identified infection	50	6	47	6	1.07	0.32, 3.59	1.19	0.35, 4.03
Intrauterine growth retardation								
Malaria only	66	9	63	9	0.96	0.35, 2.63	1.01	0.35, 2.89
Malaria and NG and/or CT	18	3	9	2	1.47	0.18, 11.72	0.64	0.07, 5.74
Malaria and TV and/or BV	86	9	96	18	1.52	0.63, 3.65	1.54	0.61, 3.88
Syphilis and any other infection(s)	7	0	10	2	1.31	0.09, Inf	1.31	0.09, Inf
NG and/or CT only	12	0	2	0	N/A	-	N/A	-
TV and/or BV only	71	8	53	8	1.15	0.40, 3.36	1.28	0.42, 3.89
No identified infection	50	5	47	4	0.84	0.21, 3.38	0.88	0.21, 3.70

CIs that do not overlap the null value of OR = 1 are shown in bold. Syphilis and coinfection describes pregnant women who tested positive for syphilis using rapid plasma reagin assays and were also infected with malaria and/or another curable sexually transmitted or reproductive tract infection. NA (not applicable) is used where there are no observations in the reference group (0–1 dose). Inf (infinity) is used where the subsample of outcomes observed is too small to produce an outer limit with certainty.

Abbreviations: BV, bacterial vaginosis; CI, confidence interval; CT, *Chlamydia trachomatis*; IPTp-SP, intermittent preventive treatment of malaria in pregnancy using sulfadoxine-pyrimethamine; NG, *Neisseria gonorrhoeae*; OR, odds ratio; TV, *Trichomonas vaginalis*.

^a^Adjusted for sexually transmitted and reproductive tract coinfection, gravidae, and human immunodeficiency virus coinfection.

^b^Syphilis and any other infection(s) refers to any other curable sexually transmitted or reproductive tract infection and/or malaria.

## DISCUSSION

To our knowledge, we are the first to investigate the association between the number of IPTp-SP doses and malaria, curable STIs/RTIs, and birth outcomes. One of our most interesting findings was that women who received ≥2 doses and had neither malaria nor curable STIs/RTIs were more protected against any adverse birth outcome, LBW, and preterm delivery compared to recipients of 0–1 dose. It appears that no additional benefit is conferred to pregnant women against nonmalaria and non-STI/RTI causes of adverse birth outcomes when ≥3 doses are administered.

These findings may partially explain why IPTp-SP has demonstrated noninferiority against more potent antimalarial compounds in several randomized clinical trials of IPTp, particularly in studies measuring LBW and preterm birth as endpoints [20–24]. Sulfadoxine is a broad-spectrum antibiotic that likely exerts an inhibitory effect against nonmalaria causes of LBW and preterm birth [25]. SP has been used to prevent *Pneumocystis jiroveci* pneumonia and *Toxoplasma gondii* infection [26]. Sulfadoxine is pharmacologically related to sulfamethoxazole, a compound coformulated with trimethoprim for the treatment of urinary tract infections and to prevent *P. jiroveci* among HIV-infected patients [27]. Prior to the development of penicillin, sulfonamides were used to treat *N. gonorrhoeae* [28]. Based on historical use, sulfonamides could exert some effect on *C. trachomatis*, *Streptococcus pyogenes*, *Streptococcus pneumoniae*, and *Haemophilus influenzae*, among many other pathogens [29]. Sulfonamides have also been used to treat *Gardnerella vaginalis* [30], a bacterium that is commonly found in high concentrations among women with bacterial vaginosis. Thus, even if sulfadoxine is only partially protective against a broad spectrum of gram-positive and gram-negative bacteria, dosing at each scheduled ANC visit from the second trimester to delivery may be sufficient to curb bacterial densities, thus directly reducing the incidence of adverse birth outcomes. It is possible, as well, that reduced bacterial densities produced by sulfadoxine exposure also inhibit maternal inflammatory responses to infections that are known to trigger preterm birth [31].

Although we found no statistically significant differences across exposure groups in the baseline characteristics specific to malaria infection and curable STIs/RTIs, our analyses did produce some notable imbalances in subgroups as reflected by some *P* values. However, confounding is not inherently produced by imbalances in group sizes. Rather, confounding is determined by observing the difference between crude ORs and adjusted ORs specific to a variable of interest [19]. We identified the following 3 confounders in this process: hypertension at enrollment or delivery (change in crude OR = 13.39%) in the stillbirth analysis (Supplementary Table 3); prior preterm birth (change in crude OR = 14.04%) in the preterm delivery analysis (Supplementary Table 5), and prior miscarriage (change in crude OR = 25.73%) in the IUGR analysis (Supplementary Table 6).

There were 3 statistically significant differences between women who were given 0–1 dose vs ≥2 doses, as follows: the wealth quintiles (*P* = .048), sex of the baby (*P* = .008), and malaria infection (*P* = .047; Table 1). Differences in wealth may not have had a consequential effect on outcomes because the imbalance straddles the 2 exposure groups; the lowest (poorest) quintile has proportionately more women from the ≥2 dose group; the second and third quintiles have more women from the 0–1 dose group; the fourth quintile has more women from the ≥ 2 dose group; and the highest (wealthiest) quintile has near equivalent representation from the 2 dose group. Regarding sex of the baby, there were fewer males born than females in the 0–1 dose group, 38.1% males (n = 48) and 61.9% females (n = 78) relative to 51.4% (n = 303) males and 48.6% (n = 287) females born to mothers who received ≥2 doses. However, this difference is unlikely to have influenced the protective effect of IPTp-SP. As for malaria infection at enrollment, 10% more women were parasitemic and went on to receive ≥2 doses of IPTp-SP compared to recipients of 0–1 dose (59.3% [n = 346] vs 49.2% [n = 62]; *P* = .047). This finding is consistent with the epidemiology of malaria in pregnancy and can be expected from the standpoint of health service delivery. Among women enrolled between 8 and 13 gestational weeks, 36.1% (95% CI, 22.5, 52.5) had peripheral parasitemia; these same women went onto receive a mean of 2.6 doses of IPTp-SP over the course of their pregnancies. However, peripheral malaria was lower (33.5%; 95% CI, 29.1, 38.3) among participants who were between 14 and 20 gestational weeks at the time of their enrollment and subsequently received a mean of 2.4 doses of IPTp-SP during their pregnancies. These 2 trends of parasitemia and the number of IPTp-SP doses continued downward when women who were between 21 and 26 gestational weeks were considered and 27 to 31 gestational weeks at their time of enrollment. These two trends of parasitemia and the number of IPTp-SP doses continued downward when comparing women at enrollment who were between 21 and 26 gestational weeks versus 27 to 31 gestational weeks; peripheral parasitemia was 31.7% (95% CI, 27.5, 36.2) and 27.6% (95% CI, 21.9, 34.2), and the mean number of IPTp-SP doses administered until delivery was 2.2 and 1.8, respectively. In our study, parasite load was highest among women enrolled at gestational week 15, with a mean density of 4752 parasites per microliter of blood. This peak of parasite density is also consistent with the epidemiology of malaria infection during pregnancy; prior reports suggest that peripheral parasitemia peaks between weeks 9 and 16 and tapers to term [32, 33]. Thus, our results confirm that pregnant women who seek ANC services earlier in their pregnancies are more likely to have a malaria infection; they are also more likely to receive more doses of IPTp-SP during pregnancy for having engaged the health system earlier in pregnancy.

Our last notable imbalance was in the proportion of pregnant women from households where indoor residual spraying had been applied in the previous 12 months (*P* = .002). This was applicable to 17.1% (n = 51) of women in the 2 dose group compared to 28.4% (n = 76) in the ≥3 dose group (Supplementary Table 1). Although indoor residual spraying is known to protect against malaria infection, this did not translate into reduced parasitemia at enrollment, as measured by polymerase chain reaction; 56.1% (n = 171) of women in the 2 dose group had malaria infections, whereas 62.9% (n = 175) in the ≥3 doses group were parasitemic.

As a nonrandomized observational study, we can only conclude that our results reflect associations between the exposure to IPTp-SP doses during ANC visits and birth outcomes and the actual mechanisms of action are unknown. Nevertheless, these observations are biologically plausible and compelling. In fact, because only 6 of the 126 women in the 0–1 dose group received 0 doses in our study, the protective effect of IPTp-SP against adverse birth outcomes related to malaria and curable STIs/RTIs is likely higher than we observed.

Our study also illustrates the importance of improving syphilis screening and treatment. Results from RPR assays were not available on the same day as sample collection. Thus, Ministry staff hand-deliver test results to RPR-positive pregnant women at their homes 3 days later and encourage patients to return to the health center with their partners for treatment. Facility staff make another home visit if RPR-positive women have not sought treatment within 4 weeks. Despite these efforts, only 64.1% (n = 52) of women received appropriate treatment. Use of rapid point-of-care tests for syphilis would allow for same-day screening and treatment [34].

It is important to consider our results in the context of the current research agenda. We know that dihydroartemisinin–piperaquine, when administered as IPTp, is superior to SP against the incidence of clinical malaria during pregnancy and the risk of maternal anemia at delivery [24]. Consequently, SP is suboptimal for preventing and clearing malaria infection in pregnant women. However, our results suggest that candidate replacements for SP should offer protection that extends beyond malaria infection to include curable STIs/RTIs. A systematic review and metaanalysis found that the prevalence of curable STIs/RTIs was similar, if not higher, when considered collectively than malaria infection among pregnant women attending ANC facilities in sub-Saharan Africa [35]. Pooled estimates for eastern and southern Africa were as follows: syphilis, 4.5% (3.9%–5.1%); *N. gonorrhoeae*, 3.7% (2.8%–4.6%); *C. trachomatis*, 6.9% (5.1%–8.6%); *T. vaginalis*, 29.1% (20.9%–37.2%); bacterial vaginosis, 50.8% (43.3%–58.4%); peripheral malaria, 32.0% (25.9%–38.0%); and placental malaria, 25.8% (19.7%–31.9%). Depending on the specific STI/RTI, the association with poor birth outcomes may not be as great as malaria infection.

Alternative therapies for use in IPTp must offer broad-spectrum protection against malaria and curable STIs/RTIs. A combination such as dihydroartemisinin–piperaquine plus azithromycin may address this public health need and warrants investigation. Azithromycin has preventive and curative effects against syphilis, *N. gonorrhoeae*, *C. trachomatis*, and potentially *T. vaginalis* [36]. Management of bacterial vaginosis in pregnancy may be more difficult. Authors of a recent systematic review found strong evidence against the use of metronidazole to reduce the risk of preterm birth. Metronidazole treatment among women in low-risk pregnancies appeared to increase preterm births (risk ratio, 1.11; 95% CI, 0.93, 1.34) and offered no effect in high-risk pregnancies (risk ratio, 0.96; 95% CI, 0.78, 1.18). In contrast, clindamycin reduced the risk of preterm birth by 13% (risk ratio, 0.87; 95% CI, 0.73–1.05) [37], is safe in pregnancy, is well tolerated, and has been used as monotherapy for the treatment of uncomplicated *Plasmodium falciparum* infection [38]. There is a need to conduct longitudinal studies to investigate the potential protective effect of treatments that involve dihydroartemisinin–piperaquine plus azithromycin and/or clindamycin that also involve the use of postpartum anthropometry to measure reductions in infant stunting [39].

## CONCLUSIONS

In this prospective cohort study of pregnant women in Zambia, the administration of ≥2 doses of IPTp-SP compared to 0–1 dose appeared to protect against adverse birth outcomes; ≥3 doses was associated with still greater protection. This dose-response relationship was significant in reducing the odds of preterm delivery. Additional research, including longitudinal studies, are needed to improve our understanding of the potential for IPTp-SP to protect against STIs/RTIs and other nonmalarial causes of adverse birth outcomes.

## Supplementary Data

Supplementary materials are available at *Clinical Infectious Diseases* online. Consisting of data provided by the authors to benefit the reader, the posted materials are not copyedited and are the sole responsibility of the authors, so questions or comments should be addressed to the corresponding author.

## Supplementary Material

Supplementary_Table_1_22_December_2016_84725R1

Supplementary_Table_2_22_December_2016_84725R1

Supplementary_Table_3_22_December_2016_84725R1

Supplementary_Table_4_22_December_2016_84725R1

Supplementary_Table_5_22_December_2016_84725R1

Supplementary_Table_6_22_December_2016_84725R1

## Notes

***Potential conflicts of interest.*** All authors: No potential conflicts of interest. All authors have submitted the ICMJE Form for Disclosure of Potential Conflicts of Interest. Conflicts that the editors consider relevant to the content of the manuscript have been disclosed.
