# Supplementary material for: Sulfadoxine-Pyrimethamine Exhibits Dose-Response Protection Against Adverse Birth Outcomes Related to Malaria and Sexually Transmitted and Reproductive Tract Infections
Source: Clin Infect Dis. 2017 Mar 2;64(8):1043–51. doi: 10.1093/cid/cix026 (PMC5399940; doi:10.1093/cid/cix026)
Supplement: Supplementary_Table_1_22_December_2016_84725R1 [file cix026_suppl_Supplementary_Table_1_22_December_2016_84725R1.docx]

| **Supplementary Table 1: Participant characteristics by exposure to 2 doses versus > 3 doses of IPTp-SP** | | | | | |
| --- | --- | --- | --- | --- | --- |
|  | **Doses of SP received – number (%)** | | | |  |
|  | **2 doses** | | **> 3 doses** | |  |
| *Characteristics at enrolment* | **n = 310** | | **n = 280** | | ***P*-value** |
| **Age of participants** |  |  |  |  | 0.521 |
| Mean (SD)***** | 25.6 | (6.4) | 25.2 | (6.4) |  |
| Median (IQR)***** | 25.0 | (20.0, 30.0) | 24.0 | (20.0, 30.0) |  |
| **Marital status** |  |  |  |  | 0.479 |
| Single | 61 | (19.7) | 62 | (22.1) |  |
| Married, divorced/separated or widowed | 249 | (80.3) | 218 | (77.9) |  |
| **Age at sexual debut** |  |  |  |  | 0.328 |
| < 15 years of age | 21 | (6.8) | 28 | (10.0) |  |
| > 15 years of age | 241 | (77.7) | 214 | (76.4) |  |
| Unknown | 48 | (15.5) | 38 | (13.6) |  |
| **Number of lifetime sexual partners** |  |  |  |  | 0.460 |
| 1 partner | 150 | (49.0) | 122 | (43.9) |  |
| 2 partners | 83 | (27.1) | 78 | (28.1) |  |
| 3 partners | 48 | (15.7) | 46 | (16.5) |  |
| 4 or more partners | 25 | (8.2) | 32 | (11.5) |  |
| **Gravidae** |  |  |  |  | 0.936 |
| Primigravidae | 86 | (27.7) | 79 | (28.2) |  |
| Secundigravidae | 42 | (13.5) | 35 | (12.5) |  |
| Multigravidae | 182 | (58.7) | 166 | (59.3) |  |
| **Wealth Quintiles** |  |  |  |  | 0.379 |
| Lowest | 68 | (21.9) | 47 | (16.8) |  |
| Second | 58 | (18.7) | 53 | (18.9) |  |
| Middle | 59 | (19.0) | 54 | (19.3) |  |
| Fourth | 56 | (18.1) | 66 | (23.6) |  |
| Highest | 69 | (22.3) | 60 | (21.4) |  |
| **Bed net ownership** |  |  |  |  | 0.564 |
| No | 160 | (51.6) | 137 | (48.9) |  |
| Yes | 150 | (48.4) | 143 | (51.1) |  |
| **Used insecticide treated net on previous night** |  |  |  |  | 0.932 |
| No | 193 | (62.7) | 173 | (62.0) |  |
| Yes | 115 | (37.3) | 106 | (38.0) |  |
| Missing | 2 |  | 1 |  |  |
| **Indoor residual spraying in the previous 12 months** |  |  |  |  | 0.002 |
| No | 247 | (82.9) | 192 | (71.6) |  |
| Yes | 51 | (17.1) | 76 | (28.4) |  |
| Missing | 12 |  | 12 |  |  |
| **Experienced miscarriage before** |  |  |  |  | 0.470 |
| No | 193 | (86.2) | 178 | (88.6) |  |
| Yes | 31 | (13.8) | 23 | (11.4) |  |
| None reported by primigravidae | 86 |  | 79 |  |  |
| **Delivered a premature baby before** |  |  |  |  | 0.297 |
| No | 214 | (95.5) | 187 | (93.0) |  |
| Yes | 10 | (4.5) | 14 | (7.0) |  |
| Not applicable to primigravidae | 86 |  | 79 |  |  |
| **Delivered a stillborn before** |  |  |  |  | 0.737 |
| No | 205 | (91.5) | 182 | (90.5) |  |
| Yes | 19 | (8.5) | 19 | (9.5) |  |
| Not applicable to primigravidae | 86 |  | 79 |  |  |
| **HIV status** |  |  |  |  | 0.528 |
| Negative | 270 | (87.1) | 249 | (88.9) |  |
| Positive | 40 | (12.9) | 31 | (11.1) |  |
| **Malaria and curable STIs/RTIs** |  |  |  |  |  |
| Malaria (PCR diagnosis) | 171 | (56.1) | 175 | (62.9) | 0.092 |
| Syphilis (high titre) | 7 | (2.3) | 10 | (3.6) | 0.462 |
| *Neisseria gonorrhoeae* | 15 | (4.8) | 6 | (2.1) | 0.117 |
| *Chlamydia trachomatis* | 16 | (5.2) | 10 | (3.6) | 0.423 |
| *Trichomonas vaginalis* | 73 | (23.5) | 67 | (23.9) | 0.923 |
| Bacterial vaginosis | 145 | (46.8) | 132 | (47.1) | 0.934 |
|  |  |  |  |  |  |
| *Characteristics at delivery* |  |  |  |  |  |
| **Place of delivery** |  |  |  |  | 0.418 |
| Hospital | 292 | (94.2) | 259 | (92.5) |  |
| Clinic | 7 | (2.3) | 12 | (4.3) |  |
| Home | 11 | (3.5) | 9 | (3.2) |  |
| **Delivery performed by** |  |  |  |  | 0.782 |
| Doctor | 20 | (6.5) | 18 | (6.4) |  |
| Midwife | 277 | (89.4) | 247 | (88.2) |  |
| Family member | 9 | (2.9) | 8 | (2.9) |  |
| Other | 4 | (1.3) | 7 | (2.5) |  |
| **Type of labour** |  |  |  |  | 0.920 |
| Spontaneous | 292 | (97.7) | 266 | (97.1) |  |
| Induced | 4 | (1.3) | 5 | (1.8) |  |
| Augmented | 3 | (1.0) | 3 | (1.1) |  |
| **Type of delivery** |  |  |  |  | 0.870 |
| Vaginal | 290 | (93.5) | 261 | (93.2) |  |
| C-section | 20 | (6.5) | 19 | (6.8) |  |
| **Hypertension** |  |  |  |  | 0.198 |
| No | 276 | (98.9) | 230 | (97.0) |  |
| Yes | 3 | (1.1) | 7 | (3.0) |  |
| **Maternal hemoglobin** |  |  |  |  | 0.429 |
| Normal | 249 | (84.7) | 221 | (82.2) |  |
| Anaemic | 45 | (15.3) | 48 | (17.8) |  |
| **Sex of baby** |  |  |  |  | 1.000 |
| Female | 151 | (48.7) | 136 | (48.6) |  |
| Male | 159 | (51.3) | 144 | (51.4) |  |
| **Received curative treatment for malaria infection** |  |  |  |  | 0.810 |
| No | 269 | (86.8) | 239 | (86.0) |  |
| Yes | 41 | (13.2) | 39 | (14.0) |  |
| **Received curative treatment for any STI/RTI** |  |  |  |  | 0.038 |
| Untreated | 291 | (93.9) | 249 | (88.9) |  |
| Treated | 19 | (6.1) | 31 | (11.1) |  |
| * Age is shown as the median value with the interquartile range in parentheses. | | | | | |
| *P*-values are from Wilcoxon rank sum test (continuous variables) or Fisher's exact test (categorical variables).  PCR = polymerase chain reaction | | | | | |
