# Supplementary material for: Sulfadoxine-Pyrimethamine Exhibits Dose-Response Protection Against Adverse Birth Outcomes Related to Malaria and Sexually Transmitted and Reproductive Tract Infections
Source: Clin Infect Dis. 2017 Mar 2;64(8):1043–51. doi: 10.1093/cid/cix026 (PMC5399940; doi:10.1093/cid/cix026)
Supplement: Supplementary_Table_2_22_December_2016_84725R1 [file cix026_suppl_Supplementary_Table_2_22_December_2016_84725R1.docx]

| **Supplementary Table 2. Confounder analysis: Any adverse birth outcome** | | | | | | | | | | | |
| --- | --- | --- | --- | --- | --- | --- | --- | --- | --- | --- | --- |
|  | **Crude analysis** | | |  | **Adjusted analysis** | |  |  |  |  |  |
| **Potential confounding variable** | **Odds ratio** | **95% CI** | ***P*-value^4^** |  | **Odds ratio** | **95% CI** | ***P*-value^4^** |  | **% change in crude odds ratio^5^** | **P-value for homogeneity** | **Missing values^6^** |
| Gravidae | 0.57 | (0.38, 0.84) | 0.004 |  | 0.52 | (0.35, 0.77) | 0.001 |  | 8.89 | 0.302 | 0 |
| Prior preterm birth^1^ | 0.42 | (0.15, 1.19) | 0.092 |  | 0.38 | (0.13, 1.11) | 0.067 |  | 7.88 | * | 620 |
| Sex of baby | 0.57 | (0.38, 0.84) | 0.004 |  | 0.59 | (0.40, 0.87) | 0.007 |  | 3.86 | 0.240 | 0 |
| Co-infection (malaria and/or STI/RTI) | 0.57 | (0.38, 0.84) | 0.004 |  | 0.54 | (0.37, 0.81) | 0.002 |  | 3.81 | 0.125 | 0 |
| Maternal age at enrolment (years) | 0.57 | (0.38, 0.84) | 0.004 |  | 0.55 | (0.36, 0.82) | 0.003 |  | 3.55 | 0.736 | 0 |
| Prior miscarriage^1^ | 0.42 | (0.15, 1.19) | 0.092 |  | 0.40 | (0.14, 1.15) | 0.079 |  | 3.39 | 0.424 | 620 |
| Placental malaria (PCR diagnosis) | 0.58 | (0.39, 0.85) | 0.005 |  | 0.56 | (0.38, 0.83) | 0.004 |  | 2.63 | 0.853 | 7 |
| Delivery type | 0.57 | (0.38, 0.84) | 0.004 |  | 0.58 | (0.39, 0.86) | 0.006 |  | 2.38 | 0.929 | 0 |
| Treatment of malaria infection during pregnancy^2^ | 0.58 | (0.39, 0.86) | 0.006 |  | 0.57 | (0.38, 0.84) | 0.004 |  | 2.23 | 0.256 | 3 |
| Marital status | 0.57 | (0.38, 0.84) | 0.004 |  | 0.55 | (0.38, 0.81) | 0.002 |  | 2.22 | 0.001 | 0 |
| Number of lifetime sexual partners | 0.56 | (0.38, 0.83) | 0.004 |  | 0.57 | (0.39, 0.85) | 0.005 |  | 2.06 | 0.564 | 6 |
| Hypertension at enrolment or delivery | 0.54 | (0.36, 0.82) | 0.003 |  | 0.55 | (0.37, 0.83) | 0.004 |  | 1.93 | 0.213 | 86 |
| Type of personnel attending birth | 0.57 | (0.38, 0.84) | 0.004 |  | 0.58 | (0.39, 0.85) | 0.005 |  | 1.78 | 0.042 | 0 |
| HIV status | 0.57 | (0.38, 0.84) | 0.004 |  | 0.57 | (0.39, 0.85) | 0.005 |  | 1.42 | 0.946 | 0 |
| Age of sexual debut (years) | 0.57 | (0.38, 0.84) | 0.004 |  | 0.57 | (0.39, 0.85) | 0.005 |  | 1.38 | 0.243 | 0 |
| Prior stillbirth^1^ | 0.42 | (0.15, 1.19) | 0.092 |  | 0.42 | (0.14, 1.25) | 0.108 |  | 1.33 | 0.358 | 620 |
| Indoor residual spraying in preceding 12 months | 0.53 | (0.36, 0.80) | 0.002 |  | 0.53 | (0.35, 0.79) | 0.001 |  | 1.30 | 0.896 | 26 |
| Syphilis at enrolment (high titre) | 0.57 | (0.38, 0.84) | 0.004 |  | 0.56 | (0.38, 0.83) | 0.004 |  | 1.16 | 0.400 | 5 |
| Labor type | 0.57 | (0.39, 0.85) | 0.005 |  | 0.57 | (0.38, 0.84) | 0.004 |  | 1.14 | * | 15 |
| Bed net ownership | 0.57 | (0.38, 0.84) | 0.004 |  | 0.57 | (0.39, 0.85) | 0.005 |  | 1.10 | 0.750 | 0 |
| Bed net usage (on night prior to survey) | 0.56 | (0.38, 0.83) | 0.004 |  | 0.55 | (0.37, 0.82) | 0.003 |  | 1.09 | 0.826 | 3 |
| Delivery location | 0.57 | (0.38, 0.84) | 0.004 |  | 0.57 | (0.38, 0.85) | 0.005 |  | 1.05 | 0.070 | 0 |
| *Trichomonas vaginalis* co-infection (malaria and/or STI/RTI) | 0.57 | (0.38, 0.84) | 0.004 |  | 0.57 | (0.39, 0.84) | 0.004 |  | 0.89 | 0.011 | 0 |
| Wealth quintiles | 0.57 | (0.38, 0.84) | 0.004 |  | 0.56 | (0.38, 0.83) | 0.004 |  | 0.84 | 0.503 | 0 |
| Maternal hemoglobin level at delivery^3^ | 0.60 | (0.40, 0.90) | 0.012 |  | 0.60 | (0.40, 0.90) | 0.012 |  | 0.56 | 0.101 | 32 |
| Treatment of STIs/RTIs during pregnancy including syphilis | 0.76 | (0.46, 1.23) | 0.259 |  | 0.76 | (0.47, 1.23) | 0.265 |  | 0.49 | 0.250 | 258 |
| STI/RTI co-infection | 0.57 | (0.38, 0.84) | 0.004 |  | 0.57 | (0.38, 0.84) | 0.004 |  | 0.39 | 0.483 | 5 |
| *Neisseria gonorrhoeae* co-infection (malaria and/or STI/RTI) | 0.57 | (0.38, 0.84) | 0.004 |  | 0.56 | (0.38, 0.84) | 0.004 |  | 0.39 | 0.318 | 0 |
| Bacterial vaginosis and STI co-infection | 0.57 | (0.38, 0.84) | 0.004 |  | 0.57 | (0.39, 0.84) | 0.004 |  | 0.33 | 0.067 | 5 |
| Recruitment site | 0.57 | (0.38, 0.84) | 0.004 |  | 0.56 | (0.38, 0.84) | 0.004 |  | 0.24 | 0.991 | 0 |
| Treatment of STIs/RTIs during pregnancy excluding syphilis | 0.57 | (0.38, 0.84) | 0.004 |  | 0.57 | (0.38, 0.84) | 0.004 |  | 0.19 | 0.492 | 0 |
| *Chlamydia trachomatis* co-infection (malaria or STI/RTI) | 0.57 | (0.38, 0.84) | 0.004 |  | 0.57 | (0.38, 0.84) | 0.004 |  | 0.04 | 0.770 | 0 |
|  |  |  |  |  |  |  |  |  |  |  |  |
| CI = Confidence Interval  PCR = Polymerase Chain Reaction  STI = Sexually Transmitted Infection  RTI = Reproductive Tract Infection  HIV = Human Immunodeficiency Virus  ^1^ Excludes women who have not been previously pregnant  ^2^ Therapy against malaria infection (apart from IPTp) after enrolment and before delivery  ^3^ Anemia was defined as haemoglobin level < 11grams/deciliter  ^4^ Confounding is not reflected in *P-*values  ^5^ Confounding is assessed by observing the difference between the crude odds ratio and adjusted odds ratio. When there is no difference (adjusted / crude – 1) between these two estimates, the observed exposure–outcome effect is not confounded by the potential confounding variable. We considered variables *a priori* that odds ratios of IPTp-SP doses by 10% or more to be potential confounders and retained them for the multivariable model. In this table, no variables demonstrated evidence of confounding on the outcome effect ‘any adverse birth outcome’.  ^6^ Missing values were excluded from the crude odds ratio  ^*^ Insufficient events to perform stratified analysis for interaction | | | | | | | | | | | |
|  | | | | | | | | | | | |
|  | | | | | | | | | | | |
|  | | | | | | | | | | | |
|  | | | | | | | | | | | |
|  | | | | | | | | | | | |
|  | | | | | | | | | | | |
|  | | | | | | | | | | | |
|  | | | | | | | | | | | |
|  | | | | | | | | | | | |
|  | | | | | | | | | | | |
|  | | | | | | | | | | | |
|  | | | | | | | | | | | |
