# Supplementary material for: Sulfadoxine-Pyrimethamine Exhibits Dose-Response Protection Against Adverse Birth Outcomes Related to Malaria and Sexually Transmitted and Reproductive Tract Infections
Source: Clin Infect Dis. 2017 Mar 2;64(8):1043–51. doi: 10.1093/cid/cix026 (PMC5399940; doi:10.1093/cid/cix026)
Supplement: Supplementary_Table_3_22_December_2016_84725R1 [file cix026_suppl_Supplementary_Table_3_22_December_2016_84725R1.docx]

| **Supplementary Table 3. Confounder analysis: Stillbirth** | | | | | | | | | | | |
| --- | --- | --- | --- | --- | --- | --- | --- | --- | --- | --- | --- |
|  | **Crude analysis** | |  |  | **Adjusted analysis** | |  |  |  |  |  |
| **Potential confounder** | **Odds ratio** | **95% CI** | ***P*-value^4^** |  | **Odds ratio** | **95% CI** | ***P*-value^4^** |  | **% change in crude odds ratio^5^** | **P-value for homogeneity** | **Missing values^6^** |
| Hypertension at enrolment or delivery | 0.88 | (0.18, 4.21) | 0.875 |  | 1.00 | (0.19, 5.32) | 1.000 |  | 13.39 | 0.422 | 86 |
| Labor type | 0.43 | (0.13, 1.46) | 0.163 |  | 0.39 | (0.11, 1.35) | 0.122 |  | 9.96 | * | 15 |
| Treatment of malaria infection during pregnancy^2^ | 0.56 | (0.15, 2.15) | 0.392 |  | 0.51 | (0.13, 2.02) | 0.328 |  | 9.31 | 0.333 | 3 |
| Indoor residual spraying in preceding 12 months | 0.43 | (0.13, 1.46) | 0.162 |  | 0.39 | (0.11, 1.36) | 0.128 |  | 8.22 | 0.584 | 26 |
| Gravidae | 0.42 | (0.12, 1.42) | 0.149 |  | 0.39 | (0.11, 1.34) | 0.121 |  | 7.42 | 0.573 | 0 |
| Delivery location | 0.42 | (0.12, 1.42) | 0.149 |  | 0.39 | (0.11, 1.39) | 0.132 |  | 6.08 | 0.459 | 0 |
| HIV status | 0.42 | (0.12, 1.42) | 0.149 |  | 0.44 | (0.13, 1.49) | 0.175 |  | 4.98 | 0.794 | 0 |
| Wealth quintiles | 0.42 | (0.12, 1.42) | 0.149 |  | 0.44 | (0.14, 1.37) | 0.144 |  | 4.92 | 0.043 | 0 |
| Delivery type | 0.42 | (0.12, 1.42) | 0.149 |  | 0.44 | (0.13, 1.48) | 0.173 |  | 4.55 | * | 0 |
| Maternal hemoglobin level at delivery^3^ | 0.57 | (0.15, 2.17) | 0.401 |  | 0.54 | (0.14, 2.11) | 0.369 |  | 4.29 | 0.694 | 32 |
| Prior stillbirth^1^ | 0.30 | (0.07, 1.39) | 0.103 |  | 0.32 | (0.07, 1.45) | 0.117 |  | 4.20 | * | 192 |
| Age of sexual debut (years) | 0.42 | (0.12, 1.42) | 0.149 |  | 0.44 | (0.13, 1.44) | 0.161 |  | 4.15 | 0.178 | 0 |
| Placental malaria (PCR diagnosis) | 0.42 | (0.13, 1.44) | 0.155 |  | 0.44 | (0.13, 1.48) | 0.171 |  | 3.20 | 0.737 | 7 |
| Type of personnel attending birth | 0.42 | (0.12, 1.42) | 0.149 |  | 0.41 | (0.12, 1.42) | 0.145 |  | 3.09 | 0.433 | 0 |
| Bed net ownership | 0.42 | (0.12, 1.42) | 0.149 |  | 0.43 | (0.13, 1.45) | 0.161 |  | 2.96 | 0.324 | 0 |
| *Neisseria gonorrhoeae* co-infection (malaria and/or STI/RTI) | 0.42 | (0.12, 1.42) | 0.149 |  | 0.43 | (0.13, 1.46) | 0.164 |  | 2.89 | * | 0 |
| Co-infection (malaria and/or STI/RTI) | 0.42 | (0.12, 1.42) | 0.149 |  | 0.43 | (0.14, 1.36) | 0.139 |  | 2.60 | 0.045 | 0 |
| Recruitment site | 0.42 | (0.12, 1.42) | 0.149 |  | 0.43 | (0.13, 1.48) | 0.168 |  | 2.59 | 0.330 | 0 |
| *Chlamydia trachomatis* co-infection (malaria or STI/RTI) | 0.42 | (0.12, 1.42) | 0.149 |  | 0.41 | (0.12, 1.39) | 0.139 |  | 2.19 | * | 0 |
| Sex of baby | 0.42 | (0.12, 1.42) | 0.149 |  | 0.43 | (0.12, 1.48) | 0.168 |  | 1.89 | 0.673 | 0 |
| Number of lifetime sexual partners | 0.42 | (0.13, 1.43) | 0.154 |  | 0.43 | (0.13, 1.45) | 0.161 |  | 1.81 | 0.461 | 6 |
| STI/RTI co-infection | 0.42 | (0.13, 1.43) | 0.153 |  | 0.43 | (0.13, 1.43) | 0.155 |  | 1.53 | 0.101 | 5 |
| Treatment of STIs/RTIs during pregnancy including syphilis | 0.55 | (0.10, 2.88) | 0.470 |  | 0.55 | (0.11, 2.93) | 0.481 |  | 1.35 | * | 258 |
| Syphilis at enrolment (high titre) | 0.42 | (0.13, 1.43) | 0.153 |  | 0.42 | (0.13, 1.33) | 0.128 |  | 1.14 | 0.007 | 5 |
| Maternal age at enrolment (years) | 0.42 | (0.12, 1.42) | 0.149 |  | 0.42 | (0.12, 1.45) | 0.158 |  | 0.78 | 0.433 | 0 |
| Prior miscarriage^1^ | 0.30 | (0.07, 1.39) | 0.103 |  | 0.31 | (0.07, 1.39) | 0.104 |  | 0.60 | 0.337 | 192 |
| Prior preterm birth^1^ | 0.30 | (0.07, 1.39) | 0.103 |  | 0.31 | (0.07, 1.40) | 0.105 |  | 0.52 | * | 192 |
| Bed net usage (on night prior to survey) | 0.42 | (0.12, 1.43) | 0.152 |  | 0.42 | (0.12, 1.42) | 0.151 |  | 0.34 | 0.408 | 3 |
| Marital status | 0.42 | (0.12, 1.42) | 0.149 |  | 0.42 | (0.12, 1.44) | 0.155 |  | 0.19 | 0.354 | 0 |
| Bacterial vaginosis and STI co-infection | 0.42 | (0.13, 1.43) | 0.153 |  | 0.42 | (0.13, 1.43) | 0.153 |  | 0.12 | 0.372 | 5 |
| Treatment of STIs/RTIs during pregnancy excluding syphilis | 0.43 | (0.13, 1.45) | 0.161 |  | 0.43 | (0.13, 1.45) | 0.162 |  | 0.07 | * | 13 |
| *Trichomonas vaginalis* co-infection (malaria and/or STI/RTI) | 0.42 | (0.12, 1.42) | 0.149 |  | 0.42 | (0.12, 1.42) | 0.149 |  | 0.00 | 0.998 | 0 |
|  |  |  |  |  |  |  |  |  |  |  |  |
| CI = Confidence Interval  PCR = Polymerase Chain Reaction  STI = Sexually Transmitted Infection  RTI = Reproductive Tract Infection  HIV = Human Immunodeficiency Virus  ^1^ Excludes women who have not been previously pregnant  ^2^ Therapy against malaria infection (apart from IPTp) after enrolment and before delivery  ^3^ Anemia was defined as haemoglobin level < 11grams/deciliter  ^4^ Confounding is not reflected in *P-*values  ^5^ Confounding is assessed by observing the difference between the crude odds ratio and adjusted odds ratio. When there is no difference (adjusted / crude – 1) between these two estimates, the observed exposure–outcome effect is not confounded by the potential confounding variable. We considered variables *a priori* that odds ratios of IPTp-SP doses by 10% or more to be potential confounders and retained them for the multivariable model. In this table, only the variable ‘Hypertension at enrolment or delivery’ demonstrated evidence of confounding on the outcome effect ‘stillbirth’.  ^6^ Missing values were excluded from the crude odds ratio  ^*^ Insufficient events to perform stratified analysis for interaction | | | | | | | | | | | |
