# Supplementary material for: Sulfadoxine-Pyrimethamine Exhibits Dose-Response Protection Against Adverse Birth Outcomes Related to Malaria and Sexually Transmitted and Reproductive Tract Infections
Source: Clin Infect Dis. 2017 Mar 2;64(8):1043–51. doi: 10.1093/cid/cix026 (PMC5399940; doi:10.1093/cid/cix026)
Supplement: Supplementary_Table_4_22_December_2016_84725R1 [file cix026_suppl_Supplementary_Table_4_22_December_2016_84725R1.docx]

| **Supplementary Table 4. Confounder analysis: Low birthweight** | | | | | | | | | | | |
| --- | --- | --- | --- | --- | --- | --- | --- | --- | --- | --- | --- |
|  | **Crude analysis** | | |  | **Adjusted analysis** | |  |  |  |  |  |
| **Potential confounder** | **Odds ratio** | **95% CI** | ***P*-value^4^** |  | **Odds ratio** | **95% CI** | ***P*-value^4^** |  | **% change in crude odds ratio^5^** | **P-value for homogeneity** | **Missing values^6^** |
| Gravidae | 0.78 | (0.49, 1.23) | 0.279 |  | 0.69 | (0.42, 1.11) | 0.122 |  | 11.86 | 0.952 | 0 |
| Prior miscarriage^1^ | 0.68 | (0.21, 2.19) | 0.513 |  | 0.64 | (0.19, 2.14) | 0.461 |  | 6.17 | 0.623 | 620 |
| Treatment of malaria infection during pregnancy^2^ | 0.78 | (0.50, 1.23) | 0.288 |  | 0.74 | (0.47, 1.18) | 0.207 |  | 4.87 | 0.034 | 3 |
| Co-infection (malaria and/or STI/RTI) | 0.78 | (0.49, 1.23) | 0.279 |  | 0.74 | (0.47, 1.17) | 0.202 |  | 4.43 | 0.211 | 0 |
| Marital status | 0.78 | (0.49, 1.23) | 0.279 |  | 0.75 | (0.48, 1.18) | 0.210 |  | 3.72 | 0.079 | 0 |
| Placental malaria (PCR diagnosis) | 0.79 | (0.50, 1.25) | 0.310 |  | 0.77 | (0.49, 1.21) | 0.255 |  | 2.98 | 0.964 | 7 |
| Indoor residual spraying in preceding 12 months | 0.73 | (0.46, 1.15) | 0.175 |  | 0.71 | (0.45, 1.13) | 0.142 |  | 2.78 | 0.450 | 26 |
| HIV status | 0.78 | (0.49, 1.23) | 0.279 |  | 0.80 | (0.51, 1.26) | 0.331 |  | 2.51 | 0.622 | 0 |
| Sex of baby | 0.78 | (0.49, 1.23) | 0.279 |  | 0.80 | (0.51, 1.25) | 0.322 |  | 2.16 | 0.364 | 0 |
| Delivery location | 0.78 | (0.49, 1.23) | 0.279 |  | 0.79 | (0.50, 1.25) | 0.321 |  | 1.90 | 0.021 | 0 |
| Bed net ownership | 0.78 | (0.49, 1.23) | 0.279 |  | 0.79 | (0.50, 1.26) | 0.324 |  | 1.88 | 0.354 | 0 |
| Wealth quintiles | 0.78 | (0.49, 1.23) | 0.279 |  | 0.77 | (0.48, 1.21) | 0.254 |  | 1.71 | 0.980 | 0 |
| Maternal age at enrolment (years) | 0.78 | (0.49, 1.23) | 0.279 |  | 0.77 | (0.48, 1.23) | 0.271 |  | 1.65 | 0.159 | 0 |
| Number of lifetime sexual partners | 0.77 | (0.49, 1.22) | 0.265 |  | 0.78 | (0.50, 1.24) | 0.295 |  | 1.58 | 0.010 | 6 |
| Syphilis at enrolment (high titre) | 0.78 | (0.49, 1.23) | 0.281 |  | 0.77 | (0.49, 1.21) | 0.253 |  | 1.54 | * | 5 |
| Delivery type | 0.78 | (0.49, 1.23) | 0.279 |  | 0.79 | (0.50, 1.24) | 0.305 |  | 1.30 | 0.546 | 0 |
| Treatment of STIs/RTIs during pregnancy including syphilis | 1.09 | (0.60, 1.96) | 0.777 |  | 1.10 | (0.61, 1.99) | 0.752 |  | 1.07 | 0.932 | 258 |
| Bed net usage (on night prior to survey) | 0.77 | (0.49, 1.21) | 0.253 |  | 0.76 | (0.48, 1.21) | 0.242 |  | 1.06 | 0.203 | 3 |
| Age of sexual debut (years) | 0.78 | (0.49, 1.23) | 0.279 |  | 0.78 | (0.50, 1.24) | 0.295 |  | 0.81 | 0.775 | 0 |
| Labor type | 0.78 | (0.49, 1.23) | 0.283 |  | 0.77 | (0.49, 1.22) | 0.271 |  | 0.70 | * | 15 |
| *Neisseria gonorrhoeae* co-infection (malaria and/or STI/RTI) | 0.78 | (0.49, 1.23) | 0.279 |  | 0.78 | (0.50, 1.23) | 0.290 |  | 0.49 | 0.592 | 0 |
| STI/RTI co-infection | 0.78 | (0.49, 1.23) | 0.281 |  | 0.78 | (0.49, 1.22) | 0.273 |  | 0.40 | 0.693 | 5 |
| *Trichomonas vaginalis* co-infection (malaria and/or STI/RTI) | 0.78 | (0.49, 1.23) | 0.279 |  | 0.78 | (0.50, 1.22) | 0.279 |  | 0.31 | 0.035 | 0 |
| Prior preterm birth^1^ | 0.72 | (0.40, 1.29) | 0.267 |  | 0.72 | (0.41, 1.28) | 0.266 |  | 0.29 | 0.019 | 192 |
| Maternal hemoglobin level at delivery^3^ | 0.80 | (0.50, 1.28) | 0.354 |  | 0.80 | (0.50, 1.28) | 0.349 |  | 0.21 | 0.120 | 32 |
| Bacterial vaginosis and STI co-infection | 0.78 | (0.49, 1.23) | 0.281 |  | 0.78 | (0.50, 1.22) | 0.272 |  | 0.21 | 0.060 | 5 |
| *Chlamydia trachomatis* co-infection (malaria or STI/RTI) | 0.78 | (0.49, 1.23) | 0.279 |  | 0.78 | (0.49, 1.23) | 0.284 |  | 0.17 | 0.435 | 0 |
| Hypertension at enrolment or delivery | 0.76 | (0.48, 1.23) | 0.264 |  | 0.76 | (0.48, 1.23) | 0.267 |  | 0.13 | 0.847 | 86 |
| Type of personnel attending birth | 0.78 | (0.49, 1.23) | 0.279 |  | 0.78 | (0.50, 1.23) | 0.279 |  | 0.10 | 0.004 | 0 |
| Treatment of STIs/RTIs during pregnancy excluding syphilis | 0.78 | (0.49, 1.23) | 0.279 |  | 0.78 | (0.49, 1.23) | 0.283 |  | 0.08 | 0.826 | 0 |
| Recruitment site | 0.78 | (0.49, 1.23) | 0.279 |  | 0.78 | (0.50, 1.22) | 0.277 |  | 0.07 | 0.053 | 0 |
| Prior stillbirth^1^ | 0.68 | (0.21, 2.19) | 0.513 |  | 0.68 | (0.20, 2.31) | 0.531 |  | 0.02 | 0.876 | 620 |
|  |  |  |  |  |  |  |  |  |  |  |  |
| CI = Confidence Interval  PCR = Polymerase Chain Reaction  STI = Sexually Transmitted Infection  RTI = Reproductive Tract Infection  HIV = Human Immunodeficiency Virus  ^1^ Excludes women who have not been previously pregnant  ^2^ Therapy against malaria infection (apart from IPTp) after enrolment and before delivery  ^3^ Anemia was defined as haemoglobin level < 11grams/deciliter  ^4^ Confounding is not reflected in *P-*values  ^5^ Confounding is assessed by observing the difference between the crude odds ratio and adjusted odds ratio. When there is no difference (adjusted / crude – 1) between these two estimates, the observed exposure–outcome effect is not confounded by the potential confounding variable. We considered variables *a priori* that odds ratios of IPTp-SP doses by 10% or more to be potential confounders and retained them for the multivariable model. In this table, only the variable ‘gravidae’ demonstrated evidence of confounding on the outcome effect of ‘low birthweight’ and was added to the multivariable model.  ^6^ Missing values were excluded from the crude odds ratio  ^*^ Insufficient events to perform stratified analysis for interaction | | | | | | | | | | | |
