# Supplementary material for: Sulfadoxine-Pyrimethamine Exhibits Dose-Response Protection Against Adverse Birth Outcomes Related to Malaria and Sexually Transmitted and Reproductive Tract Infections
Source: Clin Infect Dis. 2017 Mar 2;64(8):1043–51. doi: 10.1093/cid/cix026 (PMC5399940; doi:10.1093/cid/cix026)
Supplement: Supplementary_Table_5_22_December_2016_84725R1 [file cix026_suppl_Supplementary_Table_5_22_December_2016_84725R1.docx]

| **Supplementary Table 5. Confounder analysis: Preterm delivery** | | | | | | | | | | | |
| --- | --- | --- | --- | --- | --- | --- | --- | --- | --- | --- | --- |
|  | **Crude analysis** | | |  | **Adjusted analysis** | |  |  |  |  |  |
| **Potential confounder** | **Odds ratio** | **95% CI** | ***P*-value^4^** |  | **Odds ratio** | **95% CI** | ***P*-value^4^** |  | **% change in crude odds ratio^5^** | **P-value for homogeneity** | **Missing values^6^** |
| Prior preterm birth^1^ | 0.35 | (0.11, 1.08) | 0.056 |  | 0.30 | (0.09, 0.96) | 0.031 |  | 14.04 | * | 620 |
| Prior miscarriage^1^ | 0.35 | (0.11, 1.08) | 0.056 |  | 0.32 | (0.10, 1.01) | 0.040 |  | 8.67 | 0.326 | 620 |
| Gravidae | 0.36 | (0.23, 0.55) | 0.000 |  | 0.34 | (0.22, 0.52) | 0.000 |  | 5.63 | 0.794 | 0 |
| Sex of baby | 0.36 | (0.23, 0.55) | 0.000 |  | 0.37 | (0.24, 0.57) | 0.000 |  | 4.25 | 0.237 | 0 |
| Maternal age at enrolment (years) | 0.36 | (0.23, 0.55) | 0.000 |  | 0.34 | (0.22, 0.53) | 0.000 |  | 3.67 | 0.285 | 0 |
| Prior stillbirth^1^ | 0.31 | (0.19, 0.51) | 0.000 |  | 0.30 | (0.18, 0.50) | 0.000 |  | 3.29 | 0.032 | 192 |
| Labor type | 0.36 | (0.24, 0.56) | 0.000 |  | 0.35 | (0.23, 0.55) | 0.000 |  | 2.43 | * | 15 |
| Delivery type | 0.36 | (0.23, 0.55) | 0.000 |  | 0.36 | (0.24, 0.56) | 0.000 |  | 2.29 | 0.336 | 0 |
| Syphilis at enrolment (high titre) | 0.36 | (0.23, 0.55) | 0.000 |  | 0.35 | (0.23, 0.54) | 0.000 |  | 2.23 | * | 5 |
| Hypertension at enrolment or delivery | 0.35 | (0.22, 0.54) | 0.000 |  | 0.36 | (0.23, 0.55) | 0.000 |  | 2.22 | 0.021 | 86 |
| Co-infection (malaria and/or STI/RTI) | 0.36 | (0.23, 0.55) | 0.000 |  | 0.35 | (0.23, 0.54) | 0.000 |  | 2.08 | 0.248 | 0 |
| Type of personnel attending birth | 0.36 | (0.23, 0.55) | 0.000 |  | 0.36 | (0.24, 0.56) | 0.000 |  | 1.87 | 0.128 | 0 |
| Number of lifetime sexual partners | 0.36 | (0.23, 0.55) | 0.000 |  | 0.36 | (0.24, 0.56) | 0.000 |  | 1.57 | 0.297 | 6 |
| *Neisseria gonorrhoeae* co-infection (malaria and/or STI/RTI) | 0.36 | (0.23, 0.55) | 0.000 |  | 0.35 | (0.23, 0.54) | 0.000 |  | 1.56 | 0.331 | 0 |
| Age of sexual debut (years) | 0.36 | (0.23, 0.55) | 0.000 |  | 0.36 | (0.24, 0.55) | 0.000 |  | 1.37 | 0.734 | 0 |
| Maternal hemoglobin level at delivery^3^ | 0.37 | (0.24, 0.57) | 0.000 |  | 0.37 | (0.24, 0.57) | 0.000 |  | 1.35 | 0.212 | 32 |
| Marital status | 0.36 | (0.23, 0.55) | 0.000 |  | 0.35 | (0.23, 0.54) | 0.000 |  | 1.29 | 0.038 | 0 |
| Placental malaria (PCR diagnosis) | 0.36 | (0.24, 0.55) | 0.000 |  | 0.36 | (0.23, 0.55) | 0.000 |  | 1.10 | 0.955 | 7 |
| Bed net ownership | 0.36 | (0.23, 0.55) | 0.000 |  | 0.36 | (0.23, 0.55) | 0.000 |  | 1.07 | 0.659 | 0 |
| STI/RTI co-infection | 0.36 | (0.23, 0.55) | 0.000 |  | 0.36 | (0.23, 0.55) | 0.000 |  | 0.91 | 0.984 | 5 |
| *Trichomonas vaginalis* co-infection (malaria and/or STI/RTI) | 0.36 | (0.23, 0.55) | 0.000 |  | 0.36 | (0.23, 0.55) | 0.000 |  | 0.89 | 0.053 | 0 |
| HIV status | 0.36 | (0.23, 0.55) | 0.000 |  | 0.36 | (0.23, 0.55) | 0.000 |  | 0.84 | 0.805 | 0 |
| Wealth quintiles | 0.36 | (0.23, 0.55) | 0.000 |  | 0.36 | (0.23, 0.55) | 0.000 |  | 0.70 | 0.461 | 0 |
| Recruitment site | 0.36 | (0.23, 0.55) | 0.000 |  | 0.35 | (0.23, 0.54) | 0.000 |  | 0.66 | 0.316 | 0 |
| Indoor residual spraying in preceding 12 months | 0.32 | (0.21, 0.50) | 0.000 |  | 0.33 | (0.21, 0.50) | 0.000 |  | 0.57 | 0.450 | 26 |
| Bed net usage (on night prior to survey) | 0.36 | (0.23, 0.55) | 0.000 |  | 0.36 | (0.23, 0.55) | 0.000 |  | 0.57 | 0.894 | 3 |
| Delivery location | 0.36 | (0.23, 0.55) | 0.000 |  | 0.35 | (0.23, 0.55) | 0.000 |  | 0.49 | 0.089 | 0 |
| Treatment of STIs/RTIs during pregnancy excluding syphilis | 0.36 | (0.23, 0.55) | 0.000 |  | 0.36 | (0.23, 0.55) | 0.000 |  | 0.18 | 0.623 | 0 |
| Bacterial vaginosis and STI co-infection | 0.36 | (0.23, 0.55) | 0.000 |  | 0.36 | (0.23, 0.55) | 0.000 |  | 0.18 | 0.168 | 5 |
| *Chlamydia trachomatis* co-infection (malaria or STI/RTI) | 0.36 | (0.23, 0.55) | 0.000 |  | 0.36 | (0.23, 0.55) | 0.000 |  | 0.09 | 0.903 | 0 |
| Treatment of STIs/RTIs during pregnancy including syphilis | 0.46 | (0.27, 0.77) | 0.003 |  | 0.46 | (0.27, 0.77) | 0.003 |  | 0.09 | 0.384 | 258 |
| Treatment of malaria infection during pregnancy^2^ | 0.36 | (0.23, 0.55) | 0.000 |  | 0.36 | (0.23, 0.55) | 0.000 |  | 0.05 | 0.172 | 3 |
|  |  |  |  |  |  |  |  |  |  |  |  |
| CI = Confidence Interval  PCR = Polymerase Chain Reaction  STI = Sexually Transmitted Infection  RTI = Reproductive Tract Infection  HIV = Human Immunodeficiency Virus  ^1^ Excludes women who have not been previously pregnant  ^2^ Therapy against malaria infection (apart from IPTp) after enrolment and before delivery  ^3^ Anemia was defined as haemoglobin level < 11grams/deciliter  ^4^ Confounding is not reflected in *P-*values  ^5^ Confounding is assessed by observing the difference between the crude odds ratio and adjusted odds ratio. When there is no difference (adjusted / crude – 1) between these two estimates, the observed exposure–outcome effect is not confounded by the potential confounding variable. We considered variables *a priori* that odds ratios of IPTp-SP doses by 10% or more to be potential confounders and retained them for the multivariable model. In this table, only the variable ‘prior preterm birth’ demonstrated evidence of confounding on the outcome effect of ‘preterm delivery’ and was added to the multivariable model.  ^6^ Missing values were excluded from the crude odds ratio  ^*^ Insufficient events to perform stratified analysis for interaction | | | | | | | | | | | |
