# Supplementary material for: Sulfadoxine-Pyrimethamine Exhibits Dose-Response Protection Against Adverse Birth Outcomes Related to Malaria and Sexually Transmitted and Reproductive Tract Infections
Source: Clin Infect Dis. 2017 Mar 2;64(8):1043–51. doi: 10.1093/cid/cix026 (PMC5399940; doi:10.1093/cid/cix026)
Supplement: Supplementary_Table_6_22_December_2016_84725R1 [file cix026_suppl_Supplementary_Table_6_22_December_2016_84725R1.docx]

| **Supplementary Table 6. Confounder analysis: Intrauterine growth retardation** | | | | | | | | | | | |
| --- | --- | --- | --- | --- | --- | --- | --- | --- | --- | --- | --- |
|  | **Crude analysis** | | |  | **Adjusted analysis** | |  |  |  |  |  |
| **Potential confounder** | **Odds ratio** | **95% CI** | ***P*-value^4^** |  | **Odds ratio** | **95% CI** | ***P*-value^4^** |  | **% change in crude odds ratio^5^** | **P-value for homogeneity** | **Missing values^6^** |
| Prior miscarriage^1^ | 0.71 | (0.13, 3.92) | 0.697 |  | 0.90 | (0.13, 6.01) | 0.912 |  | 25.73 | 0.938 | 620 |
| Hypertension at enrolment or delivery | 1.55 | (0.68, 3.56) | 0.295 |  | 1.68 | (0.72, 3.94) | 0.228 |  | 8.25 | 0.883 | 86 |
| Gravidae | 1.85 | (0.82, 4.19) | 0.134 |  | 1.70 | (0.75, 3.87) | 0.198 |  | 7.89 | 0.458 | 0 |
| Maternal age at enrolment (years) | 1.85 | (0.82, 4.19) | 0.134 |  | 1.72 | (0.75, 3.90) | 0.192 |  | 7.28 | 0.419 | 0 |
| Number of lifetime sexual partners | 1.82 | (0.80, 4.12) | 0.148 |  | 1.91 | (0.83, 4.43) | 0.124 |  | 5.34 | 0.332 | 6 |
| Treatment of STIs/RTIs during pregnancy including syphilis | 3.19 | (0.95, 10.74) | 0.048 |  | 3.32 | (0.98, 11.29) | 0.042 |  | 4.11 | 0.724 | 258 |
| Placental malaria (PCR diagnosis) | 1.88 | (0.83, 4.27) | 0.123 |  | 1.81 | (0.79, 4.11) | 0.153 |  | 4.10 | 0.981 | 7 |
| Co-infection (malaria and/or STI/RTI) | 1.85 | (0.82, 4.19) | 0.134 |  | 1.78 | (0.79, 4.02) | 0.161 |  | 3.93 | 0.348 | 0 |
| Prior stillbirth^1^ | 0.71 | (0.13, 3.92) | 0.697 |  | 0.74 | (0.13, 4.07) | 0.729 |  | 3.70 | * | 620 |
| Treatment of malaria infection during pregnancy^2^ | 1.86 | (0.82, 4.21) | 0.131 |  | 1.79 | (0.79, 4.07) | 0.157 |  | 3.62 | 0.459 | 3 |
| Bed net ownership | 1.85 | (0.82, 4.19) | 0.134 |  | 1.90 | (0.83, 4.33) | 0.122 |  | 2.51 | 0.609 | 0 |
| Indoor residual spraying in preceding 12 months | 1.83 | (0.81, 4.16) | 0.142 |  | 1.79 | (0.79, 4.06) | 0.161 |  | 2.50 | 0.710 | 26 |
| Sex of baby | 1.85 | (0.82, 4.19) | 0.134 |  | 1.89 | (0.84, 4.28) | 0.120 |  | 2.24 | 0.582 | 0 |
| Wealth quintiles | 1.85 | (0.82, 4.19) | 0.134 |  | 1.81 | (0.79, 4.13) | 0.151 |  | 2.05 | 0.950 | 0 |
| Marital status | 1.85 | (0.82, 4.19) | 0.134 |  | 1.81 | (0.83, 3.99) | 0.132 |  | 1.95 | 0.037 | 0 |
| Prior preterm birth^1^ | 0.71 | (0.13, 3.92) | 0.697 |  | 0.73 | (0.13, 3.99) | 0.713 |  | 1.82 | * | 620 |
| *Trichomonas vaginalis* co-infection (malaria and/or STI/RTI) | 1.85 | (0.82, 4.19) | 0.134 |  | 1.88 | (0.84, 4.21) | 0.117 |  | 1.74 | 0.171 | 0 |
| Treatment of STIs/RTIs during pregnancy excluding syphilis | 1.91 | (0.84, 4.33) | 0.116 |  | 1.94 | (0.85, 4.41) | 0.108 |  | 1.62 | 0.685 | 13 |
| Delivery location | 1.85 | (0.82, 4.19) | 0.134 |  | 1.88 | (0.83, 4.26) | 0.127 |  | 1.34 | * | 0 |
| Maternal hemoglobin level at delivery^3^ | 1.86 | (0.82, 4.23) | 0.130 |  | 1.89 | (0.83, 4.27) | 0.121 |  | 1.27 | 0.469 | 32 |
| Labor type | 1.86 | (0.82, 4.21) | 0.132 |  | 1.88 | (0.83, 4.27) | 0.125 |  | 1.27 | * | 15 |
| HIV status | 1.85 | (0.82, 4.19) | 0.134 |  | 1.87 | (0.82, 4.23) | 0.128 |  | 0.98 | 0.757 | 0 |
| *Neisseria gonorrhoeae* co-infection (malaria and/or STI/RTI) | 1.85 | (0.82, 4.19) | 0.134 |  | 1.87 | (0.82, 4.24) | 0.129 |  | 0.91 | * | 0 |
| Bed net usage (on night prior to survey) | 1.81 | (0.80, 4.10) | 0.151 |  | 1.80 | (0.79, 4.11) | 0.159 |  | 0.59 | 0.328 | 3 |
| Recruitment site | 1.85 | (0.82, 4.19) | 0.134 |  | 1.84 | (0.82, 4.14) | 0.134 |  | 0.51 | 0.058 | 0 |
| STI/RTI co-infection | 1.87 | (0.82, 4.24) | 0.128 |  | 1.86 | (0.82, 4.22) | 0.129 |  | 0.31 | 0.407 | 5 |
| *Chlamydia trachomatis* co-infection (malaria or STI/RTI) | 1.85 | (0.82, 4.19) | 0.134 |  | 1.86 | (0.82, 4.22) | 0.134 |  | 0.26 | 0.398 | 0 |
| Type of personnel attending birth | 1.85 | (0.82, 4.19) | 0.134 |  | 1.85 | (0.82, 4.17) | 0.134 |  | 0.24 | 0.057 | 0 |
| Age of sexual debut (years) | 1.85 | (0.82, 4.19) | 0.134 |  | 1.85 | (0.83, 4.17) | 0.128 |  | 0.24 | 0.333 | 0 |
| Bacterial vaginosis and STI co-infection | 1.87 | (0.82, 4.24) | 0.128 |  | 1.87 | (0.84, 4.19) | 0.121 |  | 0.16 | 0.135 | 5 |
| Syphilis at enrolment (high titre) | 1.87 | (0.82, 4.24) | 0.128 |  | 1.87 | (0.82, 4.24) | 0.129 |  | 0.10 | * | 5 |
| Delivery type | 1.85 | (0.82, 4.19) | 0.134 |  | 1.85 | (0.82, 4.16) | 0.131 |  | 0.03 | 0.087 | 0 |
|  |  |  |  |  |  |  |  |  |  |  |  |
| CI = Confidence Interval  PCR = Polymerase Chain Reaction  STI = Sexually Transmitted Infection  RTI = Reproductive Tract Infection  HIV = Human Immunodeficiency Virus  ^1^ Excludes women who have not been previously pregnant  ^2^ Therapy against malaria infection (apart from IPTp) after enrolment and before delivery  ^3^ Anemia was defined as haemoglobin level < 11grams/deciliter  ^4^ Confounding is not reflected in *P-*values  ^5^ Confounding is assessed by observing the difference between the crude odds ratio and adjusted odds ratio. When there is no difference (adjusted / crude – 1) between these two estimates, the observed exposure–outcome effect is not confounded by the potential confounding variable. We considered variables *a priori* that odds ratios of IPTp-SP doses by 10% or more to be potential confounders and retained them for the multivariable model. In this table, only the variable ‘prior miscarriage’ demonstrated evidence of confounding on the outcome effect of ‘intrauterine growth retardation’ and was added to the multivariable model.  ^6^ Missing values were excluded from the crude odds ratio  ^*^ Insufficient events to perform stratified analysis for interaction | | | | | | | | | | | |
